# Editorial: Intracellular trafficking and membrane dynamics in health and disease

**DOI:** 10.3389/fmolb.2024.1463093

**Published:** 2024-07-22

**Authors:** Andrea Conte, Cédric Delevoye, Raffaella De Pace

**Affiliations:** ^1^ Department of Molecular Medicine and Medical Biotechnology, School of Medicine and Surgery, University of Naples Federico II, Naples, Italy; ^2^ Université Paris Cité, INSERM UMR-S1151, CNRS UMR-S8253, Institut Necker Enfants Malades, Paris, France; ^3^ Neurosciences and Cellular and Structural Biology Division, Eunice Kennedy Shriver National Institute of Child Health and Human Development, National Institutes of Health, Bethesda, MD, United States

**Keywords:** intracellular trafficking, membrane dynamics, extracellular vesicle (EV), endoplasmic reticulium, GPI proteins, retinitis pigmenstosa

Intracellular trafficking is a tightly regulated process of membrane dynamics that facilitates the exchange of cargos between cellular compartments, enabling proteins, lipids, and other macromolecules to reach their subcellular destination where they perform their functions. Membrane dynamics is crucial for the biogenesis and homeostasis of organelles, and its importance in human pathophysiology is pointed out by the evidence that over 340 monogenic diseases are caused by alterations in intracellular trafficking machineries.

In recent years, our understanding of the biogenesis of organelles, their interplay, and their functional adaptation in response to extracellular environment or stress has led to place membrane dynamics and intracellular trafficking at the heart of homeostatic cellular and tissular processes. It is therefore crucial to elucidate the cellular and molecular mechanisms of membrane dynamics and intracellular trafficking and how they are affected in human physiopathology through the development of new technological approaches and experimental models.

In this Research Topic, Cao et al. provide a new example of how alterations in intracellular trafficking are crucial determinants of the pathogenesis of human diseases. In their original research article, the authors show that certain dominant mutations in the rhodopsin gene (*RHO*), which cause retinitis pigmentosa (RP), exert their pathogenic effect by sequestering the wild-type RHO receptor in endoplasmic reticulum (ER)-associated aggregates. These pathogenic mutants impair membrane trafficking and normal localization of the wild-type receptor, while favouring its ER-associated degradation (ERAD). Such mutation with dominant-negative function may partially explain *RHO*-mediated RP processes characterized by protein misfolding and ER retention.

The study of membrane trafficking can shed light on potential diagnostic and prognostic markers facilitating the identification of new potential therapeutic targets and strategies. In their case-control study, Qadri et al. used a comparative proteomic approach to identify proteins differentially expressed in serum-derived extracellular vesicles (EVs) of non-diabetic or diabetic stroke subjects. For example, EVs from patients with diabetic stroke were enriched in components functionally associated with the complement system, and depleted in those involved in blood coagulation and neuroprotection. Beyond underlining the potential of EVs proteomics in identifying biomarkers for stroke management and prevention, these results underly the importance of membrane dynamics and trafficking in EV biology and in the control of their molecular signature in health and disease.

The intracellular trafficking can also be exploited to develop drug delivery strategies for therapeutic targeting. Okafor et al.investigated the uptake of a Cell-Penetrating Peptide (CPP) that would enable the delivery of copper (Cu) for potential therapeutic purposes in neurodegenerative disorders characterized by alteration in Cu homeostasis, like Alzheimer’s disease. This Cu(II)-binding peptide would be captured and internalized by neurosecretory cells to further release bioavailable Cu and restore normal Cu metabolism. This peptide is internalized by an ATP-dependent endocytosis pathway involving endosomes positive for both Rab5 and Rab14 whose expression is required for optimal uptake. Due to their ability to transport various therapeutic molecules across cellular membranes, the modulation of CPPs intracellular trafficking represent promising avenues for the development of treatment for a wide range of pathologies, including cancer, neurodegenerative disorders and infectious diseases.

Two articles in this Research Topic focus on glycosylphosphatidylinositol-anchored proteins (GPI-APs), which are located at the outer layer of eukaryotic plasma membranes associated to membrane microdomains and lipid rafts via glycolipid anchoring. GPI-APs can exert different functions, acting as enzymes, receptors, or adhesion molecules, thus participating in signalling pathways and response to extracellular stimuli.

Lebreton et al. unveiled cell-type dependent mechanisms regulating the organization of GPI-APs in the form of membrane-associated clusters, plausibly to adapt to different cellular environments and to achieve specialized functions. In fibroblasts, the organization of GPI-AP cluster relies on the plasma membrane-associated actin and cholesterol-dependent nanodomains, whereas epithelial cells require actin-independent but Golgi-dependent cholesterol and calcium processes for the delivery of preclustered molecules en route to the cell surface. This result shed light on how different cell types differently exploit their membrane trafficking “toolbox” to modulate how macromolecules are organized at their cell surface.

In their “Hypothesis and Theory” article, Muller and Muller analyse the potential biological mechanisms and effects of intercellular exchange of GPI-APs. In addition to their extracellular and soluble release by lipolytic cleavage, certain GPI-APs are found in body fluids, associated with EVs or micelle-like complexes containing (lyso)phospholipids and cholesterol. These structures can mediate the intercellular transfer of GPI-APs, thus modulating at distance the cell metabolism by, e.g., positively regulating glycogen and lipid synthesis. Based on this evidence, the authors put forward different hypotheses on the potential roles of GPI-APs and membrane intercellular exchange through lipid “vehicles,” considering the transfer of membrane materials as a molecular mechanism for the inheritance of acquired traits, which does not rely on modifications of DNA and histones.

Finally, Robinson et al. reviewed the latest findings about the role of ER exit sites (ERES) in physiology and disease, highlighting how ERES are key hubs for the regulation of the secretory pathway, protein quality control and cellular signalling. The authors focus on the relationships between the acute regulation of ERES and protein trafficking, present potential innovative therapeutic approaches based on ERES targeting, and discuss both their translational potential, and the limitations of their concrete usage in clinical practice.

This Research Topic presents a broad range of how cells regulate their intracellular dynamics and trafficking as well as cell-cell crosstalk trough EVs secretion, in the context of microenvironment changes due to pathogenic conditions. Studying these processes at the fundamental level is necessary for understanding the mechanisms operating in healthy and pathological conditions, and is also a crucial step for the development of biocompatible and low immunogenic strategies that can improve the treatment of patients.

